# Molecular and Serological Tests for SARS-CoV-2 Detection in Indeterminate Serology: Can We Skip the Second Sample?

**DOI:** 10.3390/cimb47110919

**Published:** 2025-11-05

**Authors:** Ivo N. Sirakov, Kalina Shishkova, Stoyan Shishkov, Ivailo Alexiev

**Affiliations:** 1 Department of Medical Microbiology “Corr. Mem. Prof. Ivan Mitov, MD, DMSc”, Faculty of Medicine, Medical University of Sofia, 2 “Zdrave” Str., 1431 Sofia, Bulgaria; 2 Laboratory of Virology, Faculty of Biology, University of Sofia “St. Kl. Ohridski”, 1164 Sofia, Bulgaria; k_shishkova@biofac.uni-sofia.bg (K.S.);; 3 National Reference Laboratory of HIV, National Center of Infectious and Parasitic Diseases, 1233 Sofia, Bulgaria; ivoalexiev@yahoo.com

**Keywords:** SARS-CoV-2, diagnostic uncertainty, antigen detection, RT-PCR, indeterminate serology

## Abstract

Indeterminate serological results for SARS-CoV-2 antibodies create diagnostic uncertainty, requiring repeat testing after 14–21 days to establish seroconversion. This study evaluated whether direct viral detection methods could provide immediate diagnostic information in serum samples with indeterminate antibody results. We analyzed 163 serum samples from clinically healthy individuals collected during March–December 2020 in Bulgaria. Samples were categorized by screening ELISA (IgA/M/G) as positive (*n* = 69), negative (*n* = 47), or indeterminate (*n* = 47). All samples underwent quantitative IgG ELISA, rapid antibody tests, rapid antigen detection (viral nucleoprotein), and RT-nested PCR. Among samples with indeterminate antibody results, 27.7% (13/47; 95% CI: 15.6–42.6%) tested positive by rapid antigen detection and 12.8% (6/47; 95% CI: 4.8–25.7%) by RT-PCR. All PCR-positive samples were also antigen-positive (Cohen’s κ = 0.69). Viral detection rates showed a gradient: antibody-positive samples 30.4% (antigen) and 16.4% (PCR), indeterminate samples 27.7% and 12.8%, antibody-negative samples 10.6% and 4.3%, respectively. The algorithm we proposed and the diagnostic methods used enable the application of certain approaches to differentiate infected from uninfected clinically healthy people, in case of intermediate antibody results. Direct viral detection identified evidence of potential SARS-CoV-2 infection in more than one-quarter of sera with indeterminate antibody results. These findings suggest immediate viral detection testing may complement standard serological approaches, though clinical validation through longitudinal studies is essential before routine implementation.

## 1. Introduction

For pathogen diagnostics, in addition to routine molecular biological, serological, and virological methods, new proposals are emerging to shorten the diagnostic algorithm, compare different methods, and introduce new ones. To understand the meaning of choosing and applying one or another method in laboratory diagnostics, one should know the basic principles of structuring and working of the methods and the resulting advantages and disadvantages. All this information influences the choice of a method for conducting routine diagnostics to prove SARS-CoV-2 infection in different laboratories. Although the SARS-CoV-2 pandemic has passed, the approaches, criteria, and discussions for defining asymptomatic and presymptomatic patients are still relevant [1,2,3,4]. The definition of asymptomatic patients is an assessment of the health status at a specific time point (on the day of sampling), and for presymptomatic patients, the definition of the status is based on the appearance of symptoms in the period (on average three days) after testing [5]. After performing an ELISA for Ab detection and obtaining a doubtful result, a re-test is performed after 14–21 days.

Serological testing for SARS-CoV-2 antibodies plays a critical role in understanding infection history and immune status at both individual and population levels [6]. However, indeterminate or borderline serological results present significant diagnostic challenges, particularly in asymptomatic individuals where clinical correlation is limited [7]. These results typically fall within a “gray zone” between clearly positive and negative values, creating uncertainty that can persist for weeks while awaiting confirmatory testing. Standard protocols recommend repeat testing after 14–21 days to establish a seroconversion when the initial results are indeterminate, introducing delays that can affect clinical decision-making and infection control measures [8].

The frequency of indeterminate serological results varies depending on the assay used, population tested, and timing of sample collection relative to infection. Studies have reported indeterminate rates ranging from 5% to 30% of tested samples, representing a substantial clinical burden [9]. During the acute phase of the COVID-19 pandemic, these diagnostic delays carried particular significance for public health measures, as individuals with uncertain infection status might continue normal activities while potentially remaining infectious, or conversely, might unnecessarily restrict activities based on diagnostic uncertainty [10].

SARS-CoV-2 infection follows predictable temporal patterns where viral components typically appear before robust antibody development [11]. The pathophysiology involves initial respiratory tract infection followed by potential systemic dissemination, during which viral RNA and antigens become detectable in blood [12]. The viral nucleocapsid protein is particularly abundant, constituting approximately 1000 copies per virion, and demonstrates relative stability compared to other viral components, making it an attractive target for antigen-based detection assays [13]. Previous studies have established that SARS-CoV-2 RNA can be detected in serum during early infection stages, often correlating with disease severity and systemic viral dissemination, with detection rates of 15–60% in hospitalized patients [14,15].

The biological mechanisms underlying indeterminate serological results are complex and multifactorial [16]. These may occur during the early phases of infection when antibody production is initiated but levels remain below traditional cutoff thresholds [17]. Individual variations in immune response kinetics, influenced by factors such as age, comorbidities, and genetic background, can result in delayed or weak antibody development [18]. Additionally, cross-reactivity with endemic coronaviruses, technical factors related to sample handling or assay performance, and the natural decline of antibody levels following resolved infection may contribute to borderline results [19,20].

The concurrent presence of indeterminate antibody levels alongside detectable viral components may indicate several clinical scenarios: early infection with developing immune response, low-level persistent infection with weak antibody production, or waning immunity with reinfection [21]. The relationship between antibody levels and viral clearance is not always straightforward, as some individuals may develop detectable but non-neutralizing antibodies that fail to effectively clear viral components from circulation [22]. Understanding the frequency of viral detection in sera with indeterminate serology could inform more efficient diagnostic algorithms and reduce the uncertainty associated with borderline serological results.

The potential advantages of immediate viral detection in cases of indeterminate serology are substantial from both clinical and public health perspectives [23]. Positive results could provide rapid confirmation of infection status, enabling appropriate isolation measures and contact tracing without the 2–3 week delay associated with repeat serology. This approach could be particularly valuable in healthcare settings, where rapid decision-making about staff scheduling and patient care protocols is essential. From a healthcare system perspective, resolving diagnostic uncertainty immediately could reduce costs associated with repeat visits, additional laboratory testing, and prolonged isolation periods, while improving patient satisfaction and reducing anxiety associated with diagnostic uncertainty [24].

This study aimed to evaluate the diagnostic value of direct viral detection methods (rapid antigen testing and RT-PCR) in serum samples with indeterminate SARS-CoV-2 antibody results and to compare performance across different serological categories, providing evidence to inform future diagnostic strategies.

## 2. Materials and Methods

### 2.1. Study Design and Participants

We included 163 serum samples in the study. This cross-sectional study analyzed serum samples collected during 13 March–8 December 2020, during the first wave of SARS-CoV-2 circulation in Bulgaria. Samples were obtained from clinically healthy people—people without symptoms of an infectious disease, as part of a research project approved by the Medical Science Council of the Medical University of Sofia (Project No. 7927/19 November 2020, Contract No. D-80/4 June 2021). The purpose and criteria for obtaining the samples were described in our previous study [25]. The samples were obtained randomly at the Southwestern Hospital and SIMD Laboratory, “St. Ivan Rilski”, Sandanski. They were collected as part of routine medical examinations. The participants did not show symptoms of SARS-CoV-2 or other infectious respiratory diseases at the time of sample collection.

### 2.2. Sample Collection and Processing

Approximately 10 mL of blood was collected by venipuncture into serum separator tubes. After clotting at room temperature for 30 min, samples were centrifuged at 2000× *g* for 10 min. Serum was aliquoted into multiple cryovials and stored at −80 °C until testing (1–2 months storage time).

### 2.3. Serological Testing

#### 2.3.1. Screening ELISA

Initial screening used semi-quantitative dual recognition ELISA for IgA/M/G antibodies (INgezim COVID-19 DR, Eurofins, Madrid, Spain). Results were categorized as positive (S/P ≥ 1.1), negative (S/P < 0.9), or indeterminate (S/P 0.9–1.1) according to manufacturer’s cutoffs.

#### 2.3.2. Quantitative IgG ELISA

A subset of samples (*n* = 106, excluding indeterminate samples) underwent quantitative IgG testing (Demeditec Diagnostics GmbH, Kiel, Germany) with results expressed in Binding Antibody Units (BAU/mL).

#### 2.3.3. Rapid Antibody Tests

Immunochromatographic lateral flow assays detected IgG and IgM separately (Artron Rapid COVID-19 Antibody Test, Artron, Burnaby, BC, Canada) according to manufacturer’s instructions.

### 2.4. Direct Viral Detection Methods

#### 2.4.1. Rapid Antigen Testing

SARS-CoV-2 nucleoprotein detection used immunochromatographic assays (NADAL rapid SARS-CoV-2 Antigen Test, NADAL, Gottingen, Germany). Serum samples (50 μL) were tested both with and without manufacturer-provided buffer; no difference was observed, confirming buffer treatment was unnecessary for cell-free serum samples.

#### 2.4.2. RNA Extraction and RT-Nested PCR

Viral RNA was extracted using HiPurA Viral DNA/RNA Purification Kit (HiMedia, Thane, India). RT-nested PCR targeted the nucleocapsid gene using a two-step protocol:

The First round (RT-PCR) of the reaction was performed using Script cDNA Synthesis Kit (Jena Bioscience GmbH, Jena, Germany): 0.5 μL SCRIPT Reverse Transcriptase, 0.5 μL RNase Inhibitor, 1 μL dNTP Mix, 4 μL SCRIPT RT Buffer complete, 4 μL RNA, 8 μL RNA free water and 2 μL primers:

External primers: Ext.2019nCoV F (5′-GGCAGTAACCAGAATGGAGA-3′) and Ext.2019nCoV R (5′-CTCAGTTGCAACCCATATGAT-3′)

Product size: 335 bp

Conditions: 42 °C—10 min; 50 °C—30 min; 70 °C—10 min

The Second round (nested PCR) of the reaction was performed in a volume of 20 µL: 2 µL cDNA from the first round, 10 µL Ruby Hot Start Master 2× (Jena Bioscience GmbH, Germany), 6 µL PCR water (Jena Bioscience GmbH, Germany) and 2 µL primers:

Internal primers: intF (5′-CACCGCTCTCACTCAACAT-3′), position 28,432–28,450 and intR (5′-CATAGGGAAGTCCAGCTTCT-3′), position 28,643–28,624

Product size: 212 bp

Conditions: 30 cycles of 95 °C—10 s, 54.6 °C—20 s, 72 °C—30 s; final extension 72 °C—10 min

Primer positions were determined according to sequence MN908947.3 from NCBI, Bethesda, MD, USA. Positive control RNA (4.5 × 10^9^ copies/mL, inactivated SARS-CoV-2 USA/WA1/2020, Microbiologics, St. Cloud, MN, USA) and negative controls were included. PCR products were confirmed by 2% agarose gel electrophoresis with ethidium bromide staining and 100 bp DNA ladder.

##### Control of the Obtained RNA and PCR Products

To control the quality and quantity of the obtained RNA and PCR products, a DNA/RNA calculator (LKB Pharmacy, London, UK) and gel electrophoresis were used. Gel electrophoresis was performed with 2% agarose (Lonza, Walkersville, MD, USA), 10 ng/mL ethidium bromide (Sigma, Burlington, MA, USA), 100 mL 1 × TAE buffer and 100 bp DNA marker (Bioline, Meridian, MS, USA) at 120–150 V, 70–120 mA for 10–40 min. The result was observed on a UV transilluminator with a wavelength of 240/260 nm.

### 2.5. Statistical Analysis

Categorical variables are presented as frequencies and percentages with 95% confidence intervals using Wilson score method [26]. Group comparisons used chi-square tests or Fisher’s exact test when expected frequencies were <5. Agreement between methods was assessed using Cohen’s kappa coefficient [27]. Statistical significance was set at *p* < 0.05. Analyses were performed using STATISTICA 8 (StatSoft Inc., Tusla, OK, USA).

### 2.6. Ethical Approval

The study was conducted according to Declaration of Helsinki guidelines ([28] World Medical Association, 2013) and approved by the Institutional Review Board of Medical University of Sofia (approval N: 1139/19 April 2021). Written informed consent was obtained from all participants.

## 3. Results

### 3.1. Study Population Characteristics

A total of 163 serum samples were analyzed from clinically healthy individuals, with 161 having sufficient volume for PCR testing (two samples had insufficient volume). The study population represented individuals sampled during the first wave of SARS-CoV-2 circulation in Bulgaria, providing insight into early pandemic dynamics before widespread vaccination or variant emergence. Initial screening ELISA results showed 69 positive samples (42.3%), 47 negative samples (28.8%), and 47 indeterminate samples (28.8%). The distribution showed no significant difference between categories (*p* = 0.073, chi-square test), indicating a balanced representation across serological categories that was optimal for comparative analysis.

### 3.2. Overall Diagnostic Performance

Table 1 presents the summary results obtained using all diagnostic methods. The plotted data show significant heterogeneity in detection rates. The screening ELISA test showed the highest positivity rate (42.3%), which was expected given its role in the initial categorization of samples and the detection of the different antibody classes (IgA, IgM, IgG) [29]. When performing a quantitative IgG ELISA test, the results obtained showed a slightly higher positivity rate (49.1%) when applied to clearly positive and negative samples, suggesting an increased sensitivity for detecting IgG in particular. The rapid IgM test showed notably high positivity (49.1%) compared to rapid IgG testing (30.7%), consistent with the early appearance and persistence of IgM antibodies in some individuals [30]. Direct viral detection methods showed lower overall positivity rates: rapid antigen testing at 23.3% and RT-PCR at 19.3%, with significant differences observed between methods (*p* < 0.001, chi-square test).

### 3.3. Results by Antibody Status

#### 3.3.1. Antibody-Positive Samples (*n* = 69)

Among initially positive samples, quantitative IgG ELISA confirmed positivity in 75.4% (52/69), suggesting that approximately one-quarter had antibody levels below the quantitative assay threshold despite positive screening results. This finding highlights the complexity of antibody detection and the importance of assay selection [31]. Direct viral detection revealed substantial rates of viral component persistence: rapid antigen testing was positive in 30.4% (21/69; 95% CI: 19.6–43.0%) and RT-PCR in 16.4% (11/67; 95% CI: 8.2–27.5%).

#### 3.3.2. Antibody-Negative Samples (*n* = 47)

Samples initially negative for antibodies demonstrated predictably low but non-zero viral detection rates: rapid antigen testing was positive in 10.6% (5/47; 95% CI: 3.5–23.1%) and RT-PCR in 4.3% (2/47; 95% CI: 0.5–14.5%). These findings could represent very early infection before antibody development, individuals with impaired antibody responses, or false-negative serological results [32]. Interestingly, rapid IgM testing detected antibodies in 34.0% (16/47) of these samples, suggesting assay-specific differences in detection capabilities or the presence of low-level antibodies below the screening ELISA threshold.

#### 3.3.3. Indeterminate Samples (*n* = 47)—Primary Study Population

Table 2 presents detailed results for the indeterminate antibody samples, representing the primary study focus. The rapid antigen test demonstrated the highest positivity rate at 27.7%, significantly higher than RT-PCR at 12.8% (*p* = 0.04, chi-square test). This difference suggests that viral proteins may be more detectable than viral RNA in sera with borderline antibody responses, possibly due to protein stability or concentration differences.

### 3.4. Concordance Between Viral Detection Methods

Analysis of agreement between viral detection methods revealed strong concordance in the indeterminate samples. All 6 PCR-positive samples were also positive by antigen testing, representing 100% concordance for PCR-positive results and suggesting that antigen testing may have superior or equal sensitivity for detecting viral components in serum. The rapid antigen test identified 7 additional positive samples that were PCR-negative, indicating either higher analytical sensitivity or detection of viral proteins that persist after RNA degradation. Overall agreement between methods showed substantial concordance: Cohen’s κ = 0.69 (95% CI: 0.44–0.94), supporting the reliability of both approaches while highlighting their complementary nature.

### 3.5. Comparative Analysis Across Antibody Categories

Table 3 summarizes the virus detection rates across all antibody test categories. A clear gradient pattern was observed, with the highest detection rates in antibody-positive samples, intermediate rates in indeterminate samples, and the lowest rates in antibody-negative samples. For antigen testing, this gradient reached statistical significance (30.4% in antibody-positive, 27.7% in indeterminate, and 10.6% in antibody-negative samples; *p* = 0.038). Pairwise comparisons indicated that the difference was driven primarily by higher antigen detection in antibody-positive versus antibody-negative individuals, while antibody-positive and indeterminate groups did not differ significantly.

For PCR detection, although detection rates followed the same direction (16.4%, 12.8%, and 4.3% across the three categories), the overall difference was not statistically significant (*p* = 0.151). Similarly, combined detection by either antigen or PCR showed a non-significant trend (*p* = 0.149).

These findings suggest that indeterminate serology often reflects true biological responses rather than technical artifacts, as viral detection rates in the indeterminate group closely resembled those of antibody-positive individuals. However, only antigen testing provided statistically robust evidence for this gradient, whereas PCR and combined detection did not reach significance.

The gradient pattern (antibody-positive > indeterminate > antibody-negative) was clearly evident for antigen detection and showed a similar but non-significant trend for PCR. The similarity between viral detection rates in antibody-positive (30.4%) and indeterminate (27.7%) samples is particularly striking, suggesting that individuals with indeterminate serology may have infection characteristics similar to those with clearly positive antibody results.

#### Study Limitations

For groups with *n* < 10, only percentage values are provided.

## 4. Discussion

The detection of antibodies in the serum against SARS-CoV-2 is not in itself sufficient as a prognostic marker for the development of the disease and the manifestation of a clinical picture. This study demonstrates that direct viral detection methods identify evidence of SARS-CoV-2 infection in more than one-quarter of serum samples with indeterminate antibody results. The detection of viral components by molecular biological methods is not always evidence of the presence of infectious virions. The difference between an infectious virion from a biological perspective, capable of replicating in cell cultures, and infection at the organismal level must be considered. The 27.7% positivity rate by rapid antigen testing and 12.8% by RT-PCR in the indeterminate group represents a clinically significant finding that could inform diagnostic decision-making and potentially reduce the need for delayed confirmatory testing in a substantial proportion of cases.

The strong concordance between rapid antigen testing and RT-nested PCR (κ = 0.69), particularly the finding that all PCR-positive samples were also antigen-positive, has important practical implications for test selection and implementation [33]. Rapid antigen tests offer several advantages including speed of results (15 min versus hours), lower cost, reduced technical complexity, and the ability to perform testing in point-of-care settings without specialized equipment [34]. The superior performance of antigen testing over PCR observed across all sample categories challenges conventional assumptions about the relative sensitivity of these testing modalities and suggests that viral proteins may be more readily detectable than viral RNA in serum samples from individuals with developing or established antibody responses.

The biological mechanisms underlying these findings likely involve several interrelated factors. Viral proteins, particularly the nucleocapsid protein, are generally more stable than viral RNA and may persist in circulation longer than intact viral genomes [35]. Viral components can persist in circulation even in the presence of detectable antibodies, challenging traditional assumptions about the relationship between antibody presence and viral clearance [36]. The formation of immune complexes between viral antigens and developing antibodies may actually protect viral proteins from degradation while maintaining their detectability by antigen assays [37,38]. In the case of the Omicron variant and its characteristics, mutations in the receptor binding domain of the S protein, which are important for the course of infection and the development of the disease, do not have a negative effect on the detection of a cocktail of antibodies, as is the case with the screening ELISA method and the immunochromatographic test, which is loaded with a cocktail of antigens [39]. However, the avoidance of virus-neutralizing antibodies by the Omicron variant prevents or hinders the formation of virus-antibody complexes, which will likely make the detection of viral components (antigens and RNA) more accessible/easier and will shorten the diagnostic process according to our algorithm. Additionally, the specific analytical characteristics of the assays used may favor antigen detection over RNA detection in serum samples, particularly when viral loads are relatively low.

For antigen detection, a clear gradient was observed across antibody categories (30.4% in positive, 27.7% in indeterminate, and 10.6% in negative samples), providing statistically significant evidence for the biological relevance of these findings. PCR detection showed a similar directional pattern (16.4%, 12.8%, and 4.3%, respectively), but this trend was not statistically significant. The similarity between antigen detection rates in antibody-positive and indeterminate samples (30.4% Vs. 27.7%) remains particularly striking, suggesting that individuals with indeterminate serology may have infection characteristics similar to those with clearly positive antibody results.

The clinical interpretation of positive viral detection in sera with indeterminate antibody levels requires careful consideration of several possible scenarios [40]. First, these results may represent early acute infection where viral replication and systemic dissemination are ongoing but antibody production is just beginning to develop. Such individuals would be expected to progress to clearly positive serology over time and might represent a transmission risk to others. Second, some cases may reflect individuals with ongoing low-level infection who mount weak antibody responses due to individual variations in immune function, age-related immune senescence, or concurrent immunosuppression [41]. Third, positive viral detection might occur in individuals with waning immunity following previous infection, where antibody levels have declined to borderline ranges but viral components persist in protected compartments or as immune complexes.

From a clinical implementation perspective, the findings suggest several potential applications. Immediate viral detection testing could provide actionable diagnostic information in emergency departments, outpatient clinics, and occupational health settings where rapid decision-making about isolation and contact tracing is essential [42]. Healthcare facilities could use this approach to make immediate decisions about staff scheduling and patient care protocols without waiting for repeat serology. The ability to resolve diagnostic uncertainty in approximately 28% of indeterminate cases could significantly impact workflow efficiency and reduce the psychological burden on patients awaiting confirmatory results.

However, the cost-effectiveness of implementing routine viral detection for all indeterminate serology cases requires careful consideration. While rapid antigen tests are relatively inexpensive, the additional laboratory infrastructure, staff training, and quality assurance measures needed for systematic implementation would represent substantial healthcare system investments. Economic analyses comparing the costs of immediate viral detection testing versus standard repeat serology protocols would need to account for factors including reduced patient visits, decreased anxiety-related healthcare utilization, improved infection control outcomes, and potential prevention of secondary transmission events.

The detection of viral components in samples with clearly positive antibody results raises fundamental questions about the relationship between antibody presence and viral clearance that have broader implications beyond diagnostic algorithms [43]. Traditional immunological understanding suggests that robust antibody responses should lead to rapid viral clearance, yet this study demonstrates significant viral component persistence even in antibody-positive individuals. This finding challenges assumptions about the protective efficacy of detectable antibodies and suggests that the relationship between antibody levels, neutralizing capacity, and viral clearance is more complex than previously appreciated.

The implications for infectivity and transmission risk remain critically important unanswered questions. The detection of viral antigens or RNA in serum does not necessarily indicate the presence of infectious virus, as these components may represent degraded viral remnants, immune complexes, or viral proteins produced by infected cells rather than complete, replication-competent virions. Determining whether individuals with positive viral detection but indeterminate serology pose transmission risks would require isolation of infectious virus through cell culture studies or epidemiological investigation of transmission events, neither of which was possible in this retrospective cross-sectional study.

Several important limitations must be acknowledged when interpreting these findings and considering potential clinical applications. The cross-sectional design provides only a snapshot of viral and antibody status at a single time point, preventing assessment of the natural history of infection, clinical outcomes, or transmission events. The study population consisted entirely of clinically healthy individuals who were asymptomatic at the time of sampling, which may limit generalizability to symptomatic patients or those with known COVID-19 exposure where the clinical stakes of diagnostic uncertainty may be higher.

The temporal context of sample collection during the first pandemic wave introduces additional considerations for contemporary application [44]. The study was conducted when the original Wuhan strain was predominant, before the emergence of variants with potentially different biological characteristics including altered antigen expression, antibody recognition patterns, or persistence in circulation. Validation studies using samples from individuals infected with current circulating variants, particularly Omicron sublineages, would be essential before implementing these findings in contemporary clinical practice.

The absence of longitudinal follow-up represents perhaps the most significant limitation from a clinical validation perspective. Without knowledge of subsequent clinical course, seroconversion patterns, or transmission outcomes, the ultimate clinical significance of positive viral detection results cannot be definitively established. Some positive results may represent true early infection that would be confirmed by subsequent seroconversion and potentially associated with transmission risk, while others might represent false-positive results, cross-reactivity, or non-infectious viral persistence.

Future research priorities should focus on addressing these limitations through carefully designed prospective studies [45]. Longitudinal cohort studies with regular follow-up testing, symptom monitoring, and assessment of transmission events would be essential for establishing the clinical significance of viral detection in indeterminate serology cases. Such studies should include diverse populations across different age groups, comorbidity profiles, and immune status to ensure broad applicability of findings. Integration of viral culture studies or other measures of infectivity would help determine whether positive viral detection correlates with transmission risk, which is crucial for developing appropriate isolation and contact tracing recommendations.

The methodological approaches demonstrated in this study also have potential applications beyond SARS-CoV-2 diagnostics. The principle of using direct pathogen detection to clarify indeterminate serological results could potentially be applied to other infectious diseases where similar diagnostic challenges exist, including other respiratory viruses, hepatitis viruses, or vector-borne pathogens. The successful adaptation of rapid antigen tests for serum-based testing opens possibilities for developing integrated diagnostic platforms that combine antibody and antigen detection in single assays.

## 5. Conclusions

Direct viral detection methods identified evidence of potential SARS-CoV-2 infection in 13–28% of serum samples with indeterminate antibody results. The rapid antigen test showed higher sensitivity than RT-PCR with strong concordance between positive results. While these findings suggest that immediate viral detection testing may complement standard serological approaches in managing diagnostic uncertainty, clinical validation through prospective longitudinal studies is essential before routine implementation. These results provide preliminary evidence for developing diagnostic algorithms that might reduce time to clinical decision-making in cases of serological uncertainty. However, the relationship between viral detection in serum and clinical outcomes requires further investigation to establish whether such approaches would improve patient care in practice.

A limitation of the study is the lack of second serum samples, the reason for this being the specificity of the initial samples and the method of their selection indicated in another study of ours—from clinically healthy people to search for asymptomatic infection with SARS-CoV-2 ([25] Sirakov et al., 2024). However, the results obtained are sufficient to serve as a starting point for further studies in this direction.

## Figures and Tables

**Table 1:** Overall diagnostic results across all testing methods (*n* = 163).

Method	Total Tested	Positive *n* (%)	Negative *n* (%)	95% CI for Positivity
Screening ELISA IgA/M/G	163	69 (42.3)	94 (57.7) ^a^	34.6–50.3
Quantitative IgG ELISA	106 ^b^	52 (49.1)	54 (50.9)	39.1–59.1
Rapid IgG Test	163	50 (30.7)	113 (69.3)	23.7–38.4
Rapid IgM Test	163	80 (49.1)	83 (50.9)	41.2–57.0
Rapid Antigen Test	163	38 (23.3)	125 (76.7)	17.1–30.5
RT-nested PCR	161 ^c^	31 (19.3)	130 (80.7)	13.5–26.2

^a^ Includes 47 indeterminate results; ^b^ Excludes indeterminate screening samples; ^c^ Insufficient sample volume in 2 cases.

**Table 2:** Diagnostic results in samples with indeterminate antibody status (*n* = 47).

	Positive *n* (%)	Negative *n* (%)	95% CI for Positivity	*p*-Value ^a^
**M** **ethod**				
Rapid Antigen Test	13 (27.7)	34 (72.3)	15.6–42.6	<0.001
RT-nested PCR	6 (12.8)	41 (87.2)	4.8–25.7	<0.001
Rapid IgG Test	4 (8.5)	43 (91.5)	2.4–20.4	<0.001
Rapid IgM Test	21 (44.7)	26 (55.3)	30.2–59.9	0.271

^a^ Chi -square test comparing positive vs. negative results.

**Table 3:** Viral detection rates by initial antibody status.

Antibody Status	*n*	Antigen Positive *n* (%)	PCR Positive *n* (%)	Combined Positive ^a^ *n* (%)
Positive	69	21 (30.4)	11 (16.4) ^b^	21 (30.4)
Indeterminate	47	13 (27.7)	6 (12.8)	13 (27.7)
Negative	47	5 (10.6)	2 (4.3)	7 (14.9)
*p*-value for trend ^c^		<0.038	0.151	0.149

^a^ Positive by either antigen OR PCR; ^b^ 67 samples tested (2 insufficient volume); ^c^ Chi-square test for trend across categories.

## Data Availability

The original contributions presented in this study are included in the article material. The data presented in this study are available upon request from the corresponding author.

## Abbreviations

Table following abbreviations are used in this manuscript:

ELISA	Enzyme-Linked Immunosorbent Assay
PCR	Polymerase Chain Reaction
RT	Reverse Transcription
RNA	Ribonucleic Acid
cDNA	complementary Deoxyribonucleic acid
RT-nested PCR	Reverse Transcription-nested Polymerase Chain Reaction
